# Non-targeted and targeted metabolomics approaches to diagnosing lung cancer and predicting patient prognosis

**DOI:** 10.18632/oncotarget.11521

**Published:** 2016-08-23

**Authors:** Xiaoli Zhang, Xinyue Zhu, Caihong Wang, Haixia Zhang, Zhiming Cai

**Affiliations:** ^1^ The Affiliated Luohu Hospital of Shenzhen University, Shenzhen 518001, China; ^2^ College of Chemistry and Chemical Engineering, Lanzhou University, Lanzhou 730000, China; ^3^ Shijiazhuang Huaguang Traditional Chinese Medicine Tumor Hospital, Shijiazhuang 050000, China

**Keywords:** lung cancer, non-targeted metabolomics, targeted metabolomics, proton nuclear magnetic resonance spectroscopy, rapid resolution liquid chromatography

## Abstract

Lung cancer is the most common cause of cancer death in China. We characterized metabolic alterations in lung cancer using two analytical platforms: a non-targeted metabolic profiling strategy based on proton nuclear magnetic resonance (^1^H-NMR) spectroscopy and a targeted metabolic profiling strategy based on rapid resolution liquid chromatography (RRLC). Changes in serum metabolite levels during oncogenesis were evaluated in 25 stage I lung cancer patients and matched healthy controls. We identified 25 metabolites that were differentially regulated between the lung cancer patients and matched controls. Of those, 16 were detected using the non-targeted approach and 9 were identified using the targeted approach. Both groups of metabolites could differentiate between lung cancer patients and healthy controls with 100% sensitivity and specificity. The principal metabolic alternations in lung cancer included changes in glycolysis, lipid metabolism, choline phospholipid metabolism, one-carbon metabolism, and amino acid metabolism. The targeted metabolomics approach was more sensitive, accurate, and specific than the non-targeted metabolomics approach. However, our data suggest that both metabolomics strategies could be used to detect early-stage lung cancer and predict patient prognosis.

## INTRODUCTION

Cancer is a life-threatening disease characterized by abnormal cellular growth. The risk of cancer increases with age, exposure to environmental carcinogens, and an unhealthy lifestyle [1, 2]. Despite decades of research, cancer is still a leading cause of death. This can be explained in part by the lack of sensitive early screening tests, diagnosis at a late stage, and the metastatic behavior of tumors. The 5-year survival rate is more than 90% for patients with stage I disease. Treatment is often curative if lesions are detected at a pre-malignant stage. However, the survival rates are poor if tumors are detected at an advanced stage [3–5]. Although early diagnosis can improve survival rates, most cancer-related symptoms do not manifest until advanced stages. This leads to delays in diagnosis and treatment. Thus, new diagnostic techniques and methods for predicting prognosis are required.

Lung cancer is the leading cause of cancer-related deaths in China. There were approximately 1.8 million individuals worldwide who were diagnosed with lung cancer in 2012. Interestingly, more than one-third of these cases were diagnosed in China [6]. The survival rates for lung cancer are 56%, 34%, 10%, and 2% for stage I, II, III, and IV disease, respectively [7–8]. Over the past decade, there have been significant advances in the field of metabolomics. Metabolomics is a complementary approach to genomics and proteomics. It is a rapidly emerging field focused on comprehensive profiling of all small molecular weight metabolites in biofluids, tissues, and cells using nuclear magnetic resonance (NMR) spectroscopy, gas chromatography-mass spectrometry, and liquid chromatography-mass spectrometry [9]. Metabolomics offers unique insights into the regulation of small-molecule metabolites and the signaling pathways underlying various biological processes [10]. For example, metabolomics approaches have been used to identify cancer biomarkers and the metabolites/metabolic pathways that regulate tumor progression [11–15].

Metabolomics approaches can be targeted or non-targeted. Targeted metabolomics refers to the quantitative measurement of a select group of metabolites (e.g. amino acids, lipids, sugars, and/or fatty acids) in order to investigate specific metabolic pathways or to validate biomarkers identified using non-targeted metabolic profiling [16]. Targeted approaches require *a priori* knowledge of metabolites of interest and known compounds, are based on metabolite-specific signals, and do not achieve global coverage [17]. In contrast, non-targeted metabolomics approaches involve global profiling of the metabolome. This approach is typically employed in hypothesis-generating studies such as biomarker discovery, where comprehensive metabolite identification is generally not the goal [18]. Thus, non-targeted metabolomics often provides more information than targeted metabolomics, but targeted metabolomics typically is more quantitative.

In this study, we focused on serum free amino acids (SFAAs) and used a targeted rapid resolution liquid chromatography (RRLC)-based quantitative metabolomics approach to elucidate metabolic alterations during lung cancer progression. We also used a non-targeted proton nuclear magnetic resonance (^1^H NMR)-based metabolomics approach. The goal of this pilot investigation was to verify the ability of our metabolomics approach to identify metabolic changes during lung cancer progression. We also compared the diagnostic and predictive power of both the targeted and non-targeted approaches.

## RESULTS

### Clinical characteristics of the study population

A total of 25 patients including 15 (60.0%) men and 10 (40.0%) women (mean age 51.2 ± 10.6 years) with stage I lung cancer and 25 sex- and age-matched healthy controls (mean age 49.5 ± 8.2 years, 15 men, 10 women) were included in the metabolomics analysis. The characteristics of the patients at baseline are shown in Table 1. All patients and healthy controls were non-smokers. All patients were diagnosed with stage Ia (≤ 2 cm) or Ib (> 2 cm to ≤ 3 cm) lung cancer (9 of the cases were adenosquamous carcinoma, 12 were adenocarcinoma, and 4 were small cell lung carcinomas) by low-dose computed tomography (LDCT) and computed radiography (CR).

**Table 1:** Clinical characteristics of 25 patients with lung cancer

Subjects		Percent (%)
Gender	male	60
	female	40
Age	40 ~ 50	52
	50 ~ 60	32
	> 60	16
Pathologic type	adenosquamous carcinoma	36
	Adenocarcinomas	48
	small cell carcinomas	16
Pathologic staging	Ia	76
	Ib	24

### Non-targeted metabolomics analysis by ^1^H NMR

### ^1^H NMR analysis

**Figure 1:**
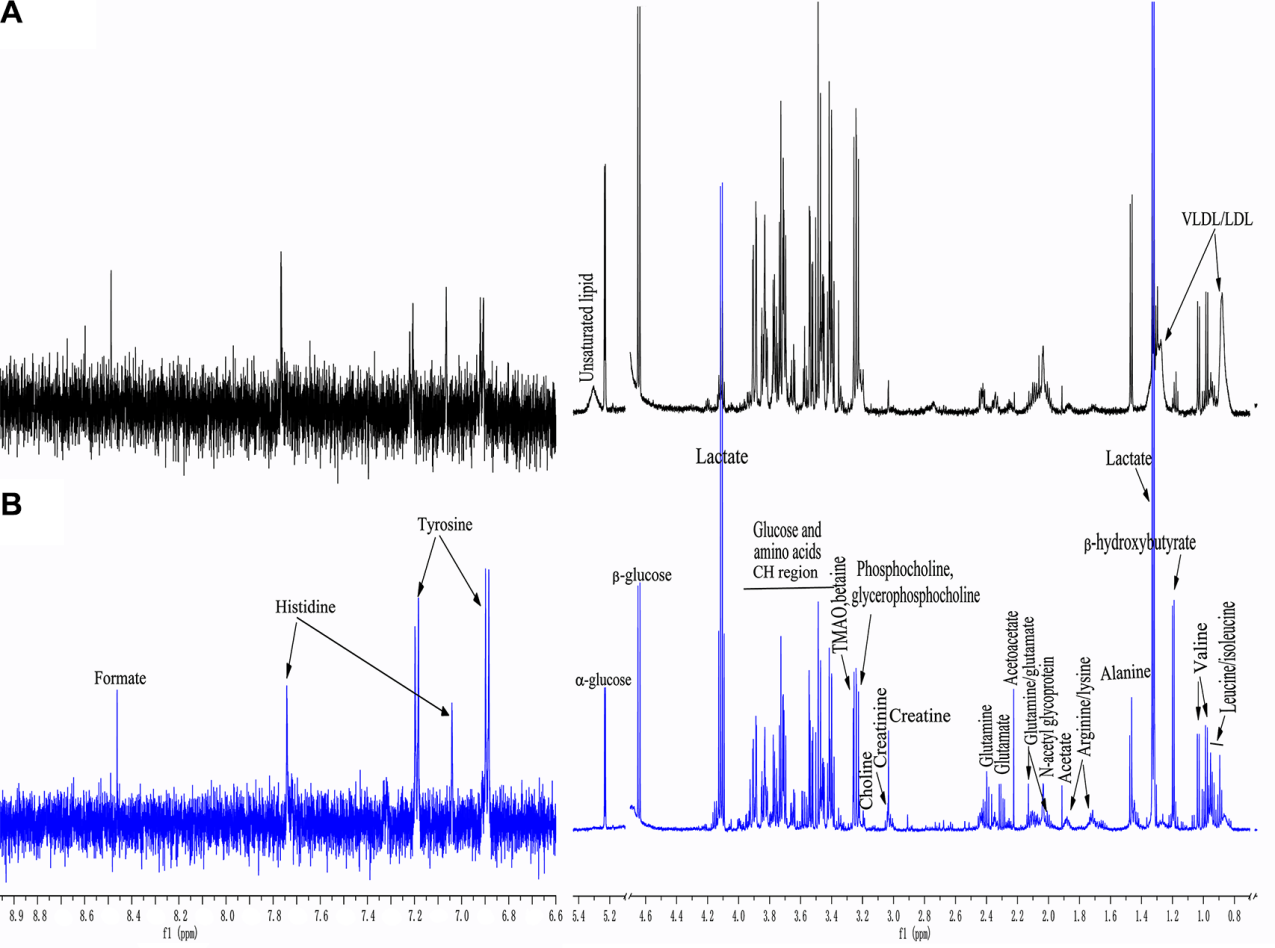
Representative ^1^H-NMR spectra of serum samples from a healthy control (A) and a lung cancer patient (B).(δ 6.6.8.9 ppm were expanded 8 times)

Representative ^1^H NMR spectra from serum samples corresponding to a healthy control and a lung cancer patient are shown in Figure 1. Metabolites were assigned according to in-house databases, published literature [19–23], and the Human Metabolome Database (HMDB, http://www.hmdb.ca/). The ^1^H NMR spectra primarily contained resonances from lipids, glucose, amino acids, and other organic acids. Choline phospholipid metabolites including choline, phosphocholine, and glycerophosphocholine, as well as ketone bodies such as β-hydroxybutyrate and acetoacetate were also detected. Other assigned metabolites included N-acetyl glycoprotein, trimethyl-amine N-oxide (TMAO) and betaine.

A comparison of the ^1^H NMR spectra between the lung cancer patients and corresponding healthy controls revealed distinct spectral changes. For example, an increase in lactate (visible at 1.33 and 4.13 ppm), ketone bodies (acetoacetate [visible at 2.23 ppm], and β-hydroxybutyrate [visible at 1.19 ppm]), and a decrease in lipids and glucose (visible at 5.23 [α-glucose] and 4.68 ppm [β-glucose], respectively) were observed. An increase in tyrosine levels and a decrease in alanine levels were also observed in the lung cancer patients compared to the healthy controls.

### Multivariate statistical analysis

**Figure 2:**
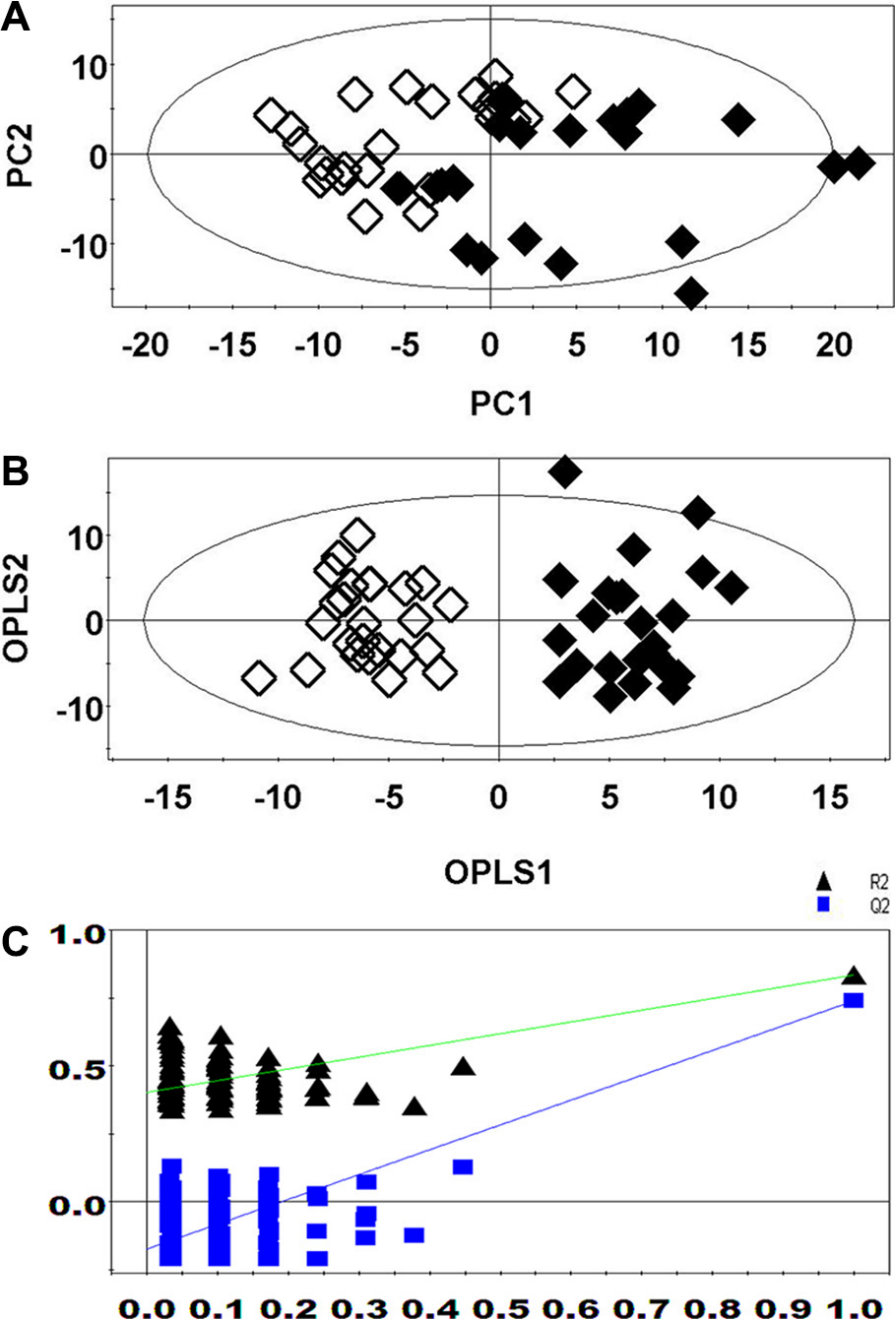
PCA (A), OPLS-DA (B), and permutation tests (C) of ^1^H NMR-based non-targeted metabolic profiling. (Healthy controls, ◆; Lung cancer patients, ◇)

PCA revealed a clear separation between the lung cancer patients and healthy controls (Figure 2A). The supervised OPLS-DA score plot (Figure 2B) also showed a separation between the lung cancer patients and healthy controls. The cumulative R^2^Y and Q^2^ were 0.891 and 0.796, respectively, when one PLS component and one orthogonal component were analyzed. No over-fitting was observed based on the results of permutation tests (Figure 2C, the R^2^ Y-intercept was 0.379 and the Q^2^-intercept was −0.171).

### Differences in metabolites between lung cancer patients and healthy controls

Variables from the OPLS-DA model with a VIP > 1 and an independent sample *t*-test (*p* < 0.05) were classified as differentially regulated metabolites that could discriminate between patients in the lung cancer and control groups. We identified 16 metabolites that were potential diagnostic biomarkers for early-stage lung cancer (Table 2). Increased levels of lactate, ketone bodies (acetoacetate and β-hydroxybutyrate), and several amino acids including glutamate, glutamine, histidine, and tyrosine were detected in lung cancer patients compared to healthy controls. Decreased levels of glucose (α- and β-glucose), lipids, unsaturated lipids, phospholipids intermediates (choline, phosphocholine, and glycerophosphocholine), TMAO, and betaine were observed in lung cancer patients compared to healthy controls.

**Table 2:** Differentially regulated metabolites identified from ^1^H NMR-based non-targeted metabolic profiling

Metabolites	Chemical shift (ppm) and multiplicity^a^	Variations versus healthy controls^b^	Metabolic pathways
LDL/VLDL	0.88(bs), 1.30(bs)	↓	Lipid metabolism
Unsaturated lipids	5.32(bs)	↓	
β-hydroxybutyrate	1.20(t)	↑	Ketogenesis, Lipid metabolism
Acetoacetate	2.25(s)	↑	
α-Glucose	5.23(d)	↓	Glycolysis
β-Glucose	4.68(d)	↓	
Lactate	1.33(d) 4.13(q)	↑	
Glutamate	2.02(m) 2.30(m)	↑	Glutamine/glutamate metabolism
Glutamine	2.10(m) 2.42(m)	↑	
Tyrosine (Tyr)	6.89(d) 7.20(d)	↑	Tyrosine metabolism
Histidine (His)	7.05(s) 7.72(s)	↑	Histidine metabolism
Choline	3.22(s)	↓	choline phospholipid metabolism
Phosphocholine	3.25(s)	↓	
Glycerophosphocholine	3.26(s)	↓	
Betaine	3.27(s)	↓	
TMAO	3.28(s)	↓	

a s, singlet; d, doublet; t, triplet; q, quartet; m, multiplet; bs, broad singlet.

b ↑ and ↓ indicate increased and decreased levels in lung cancer patients compared to healthy controls, respectively.

### Targeted metabolomics analysis by RRLC

#### RRLC analysis of SFAAs

The protocols for identification of the 23 SFAAs, including the sample preparation and RRLC analysis, were performed as described previously [24]. Validation studies of the linearity, precision, stability, and recovery of the method (Supplementary Table S1) indicated it was reliable. The concentrations of 23 SFAAs in lung cancer patients and healthy controls are shown in Supplementary Table S2.

#### Multivariate statistical analysis

**Figure 3:**
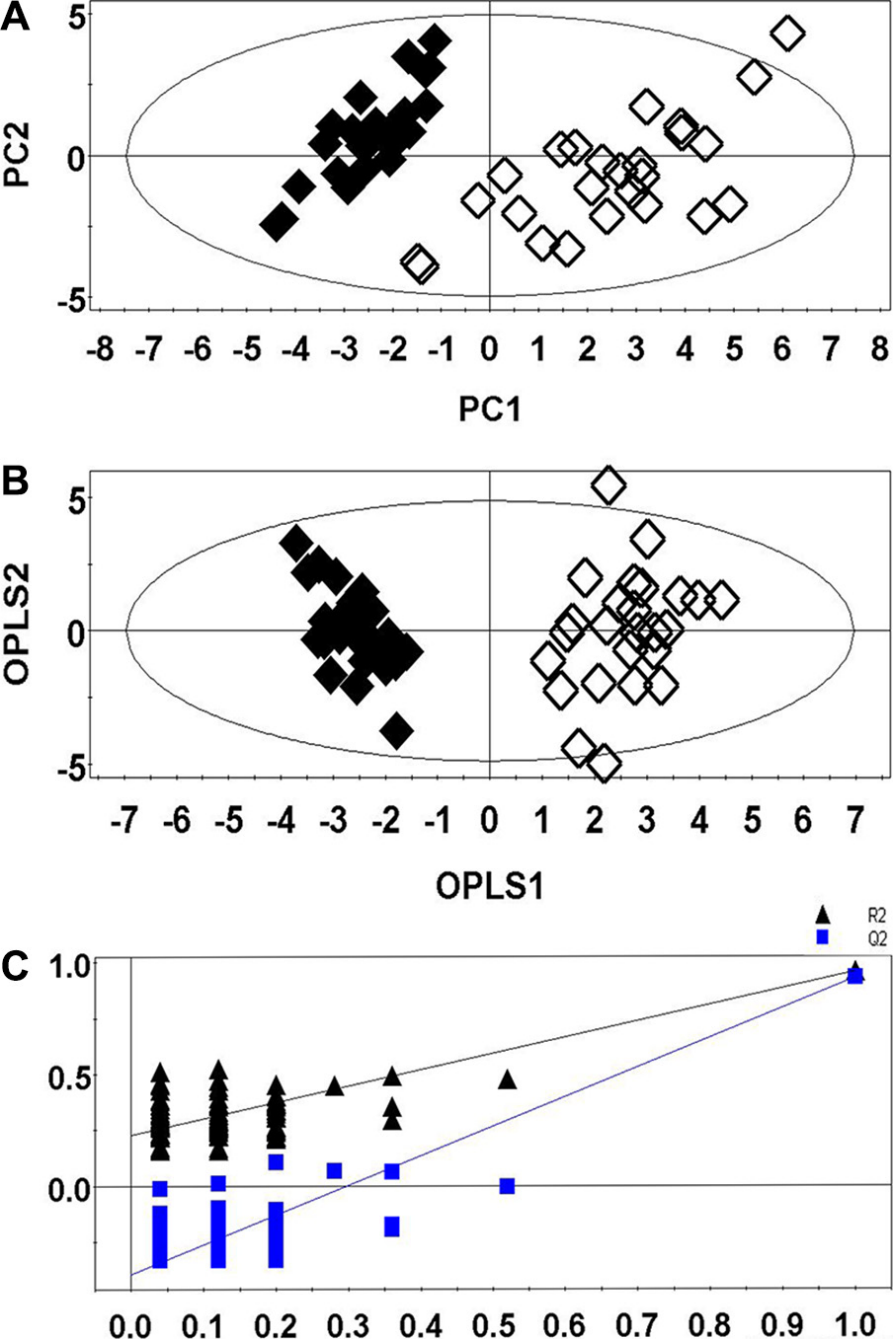
PCA (A), OPLS-DA (B), and permutation tests (C) of RRLC-based targeted metabolic profiling. (Healthy controls, ◆; Lung cancer patients, ◇)

The PCA (Figure 3A) showed a clear separation between the lung cancer and control groups. Lung cancer patients were also separated from the healthy controls by the OPLS-DA score plots (Figure 3B). Permutation tests consisting of 100 permutations demonstrated that the model was not over-fitted (Figure 3C, R^2^ Y-intercept was 0.255 and Q^2^-intercept was −0.352). CV-ANOVA indicated that the differences between the lung cancer and control groups were statistically significant (*p* = 6.75 × 10^−25^).

#### Differentially regulated amino acids between lung cancer patients and healthy controls

We selected 9 amino acids with VIP values > 1 and *p* < 0.05 as differentially regulated amino acids between lung cancer patients and healthy controls. The names of these amino acids and associated metabolic pathways are shown in Table 3. Our data indicated that the levels of aspartate, asparagine, glutamate, glutamine, cysteine, isoleucine, and leucine were higher, while the levels of methionine and tyrosine were lower, in serum samples from lung cancer patients compared to controls.

**Table 3:** Differentially regulated amino acids identified from RRLC-based targeted metabolic profiling

Metabolites	Retention time (t_R_, min)	Variations versus healthy controls^a^	Related metabolic pathways
Aspartate (Asp)	0.52	↑	Asparagine/aspartate metabolism
Asparagine (Asn)	1.95	↑	
Glutamate (Glu)	0.75	↑	Glutamine/glutamate metabolism
Glutamine (Gln)	2.09	↑	
Cysteine (Cys)	6.41	↑	One-carbon metabolism
Methionine (Met)	6.71	↓	
Isoleucine (Ile)	7.27	↑	Branched chain amino acids (BCAA) metabolism
Leucine (Leu)	7.44	↑	
Tryptophan (Trp)	7.35	↓	Tryptophan metabolism

a ↑ and ↓ indicate increased and decreased levels in lung cancer patients compared to healthy controls, respectively.

### Metabolic pathway analysis of the differentially regulated metabolites

**Figure 4:**
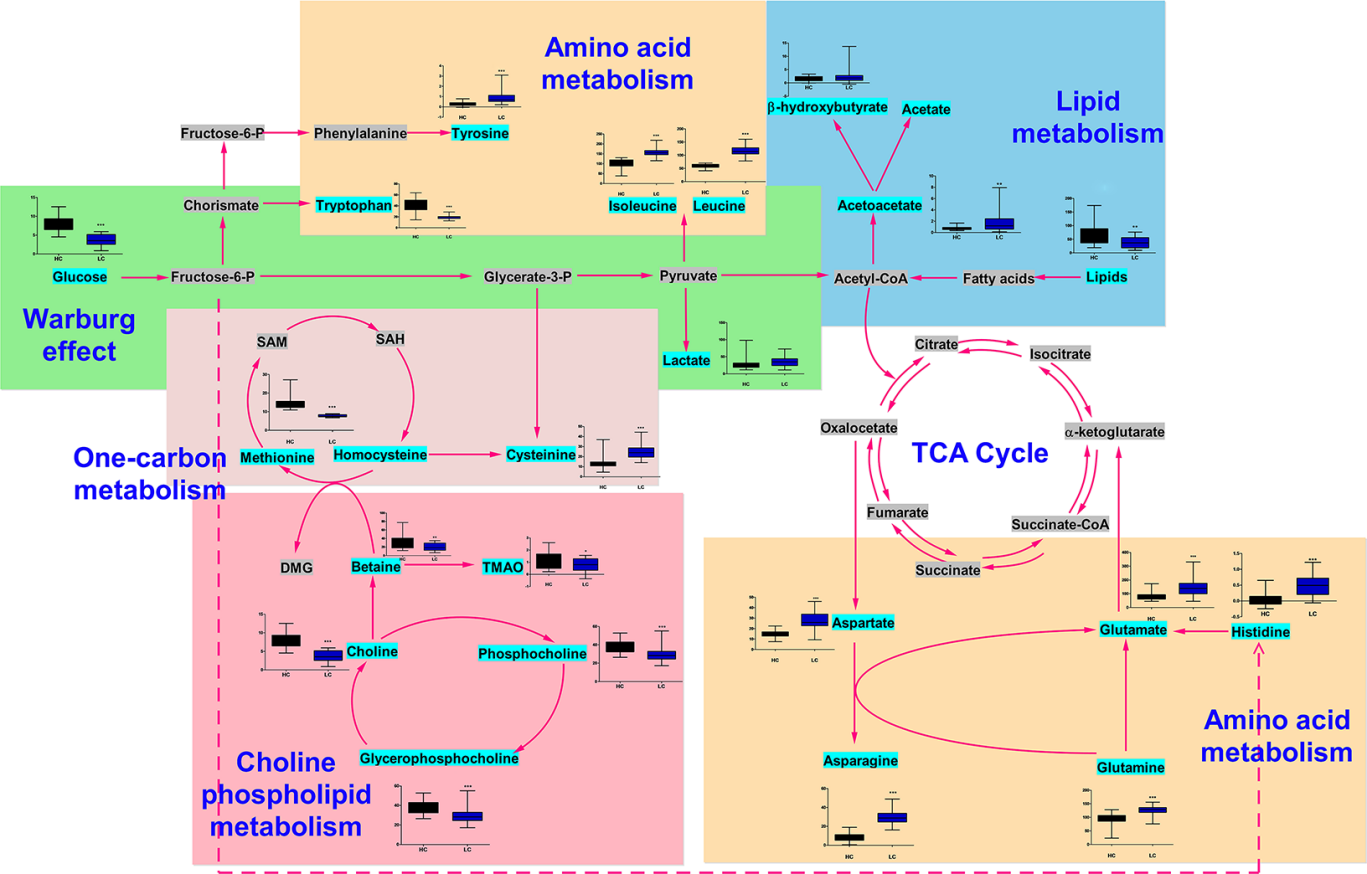
Metabolic network showing the differentially regulated metabolites The normalized contents of each metabolite are shown next to the chemical name. Black and blue bar chart showing the normalized content in the healthy control and lung cancer groups, respectively.

Based on our knowledge of the differentially regulated metabolites and an online database of metabolic pathways (Kyoto Encyclopedia of Genes and Genomes, http://www.genome.jp/kegg/), a map of lung cancer-related metabolic pathways was constructed (Figure 4). All of the differentially regulated metabolites were included in the analysis in order to obtain a global view of tumor metabolism and assess metabolic changes during lung cancer progression. The normalized levels are shown next to the chemical names. Several metabolic pathways were altered in lung cancer patients. These pathways included glycolysis (“Warburg effect”) as well as lipid, choline phospholipid (Kennedy pathway), one-carbon, and amino acid metabolism. These pathways are altered in many cancers and are associated with oncogenesis and tumor progression.

### Diagnostic accuracy of differentially regulated serum metabolites

**Figure 5:**
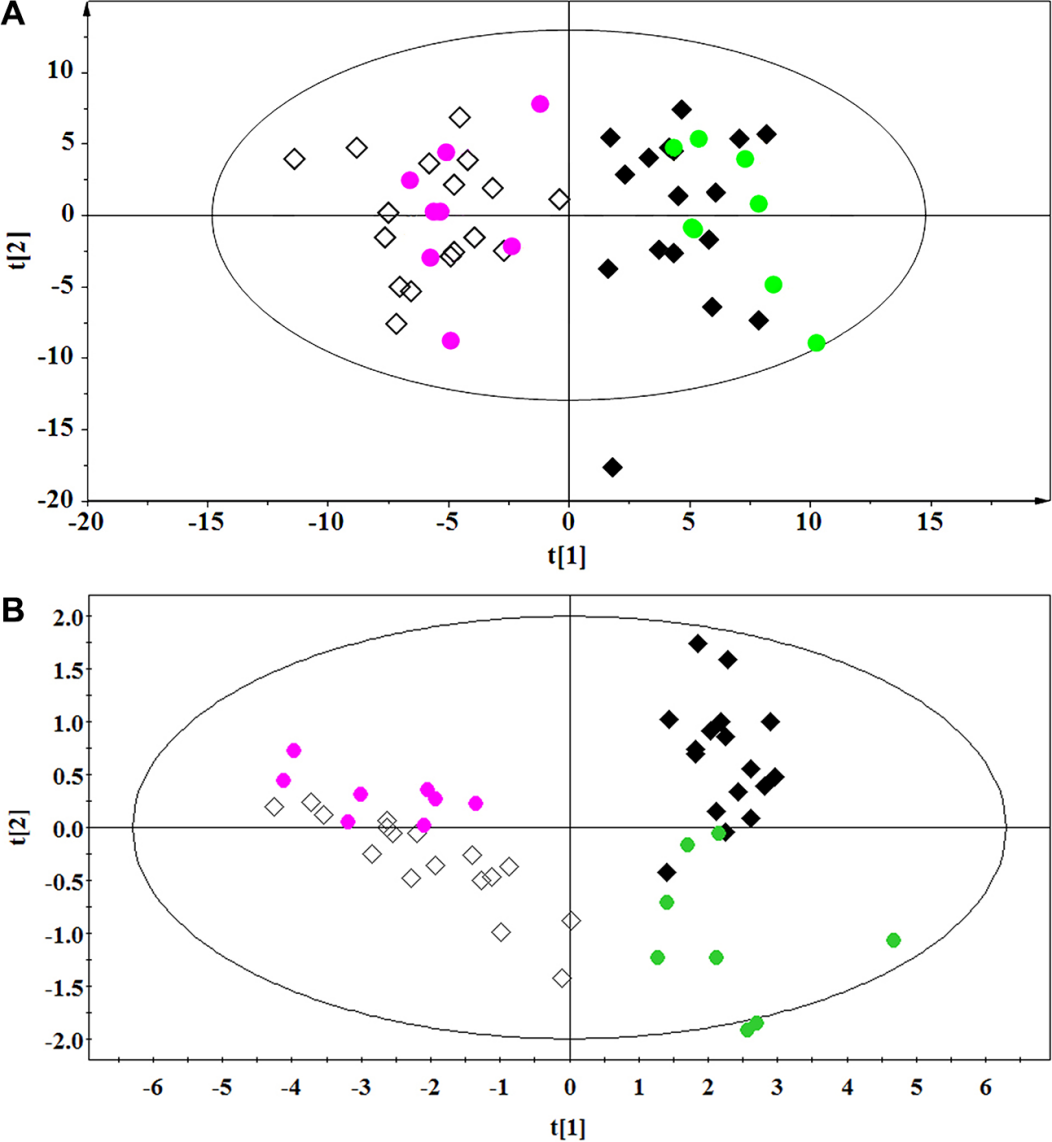
External cross-validation (**A**) ^1^H NMR-based non-targeted and (**B**) RRLC-based targeted metabolic profiling. ◆ and 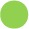, Healthy controls in the training and test sets, respectively; ◇ and 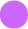, Lung cancer patients in the training and test sets, respectively.

The diagnostic accuracy of the differentially regulated metabolites, which were identified using non-targeted and targeted metabolic profiling, was investigated using the external cross-validation methods described above. The results indicated that both OPLS-DA models could correctly predict all lung cancer patients and healthy controls (100% sensitivity and specificity) (Figure 5A and 5B). Thus, non-targeted and targeted metabolomics-based approaches can be used to diagnose lung cancer.

## DISCUSSION

We evaluated the metabolomics profiles of lung cancer patients. Both ^1^H NMR-based non-targeted and RRLC-based targeted metabolic profiling revealed clear differences between lung cancer patients and healthy controls. These results suggested that both approaches could be used to investigate metabolic changes associated with early-stage lung cancer.

### Comparison between the non-targeted and targeted metabolomics approaches

We assessed whether the two different metabolomics approaches could detect changes in circulating metabolites in lung cancer patients compared to healthy controls. First, we used a non-targeted ^1^H NMR-based metabolomics approach to characterize the serum metabolic profiles of lung cancer patients and healthy controls. Non-targeted ^1^H NMR-based metabolomics analysis can be used to simultaneously measure hundreds of endogenous metabolites. It requires minimal sample preparation, allows for rapid and nondestructive sample collection, and displays high repeatability and reproducibility. We also used an RRLC-based targeted metabolomics approach to analyze SFAAs and develop a more accurate and simplified classification model. SFAA profiling is a promising approach because SFAAs have essential roles in metabolism. Additionally, the concentrations of SFAAs are influenced by metabolic variations induced by specific diseases such as cancer.

PCA and OPLS-DA were performed to determine whether the two methods could distinguish between lung cancer patients and controls. The R^2^ and Q^2^ values obtained from both the PCA and OPLS-DA demonstrated that the RRLC-based targeted metabolomics strategy had better classification and predictive ability than the ^1^H NMR-based non-targeted strategy. Metabolic profiling of serum samples using both approaches yielded a total of 25 differentially regulated metabolites (16 were identified using the non-targeted and 9 were identified using the targeted approach). In the external cross-validation, both sets of differentially regulated metabolites could accurately predict all the test samples from lung cancer patients and healthy controls, with 100% sensitivity and specificity. We determined that the targeted metabolomics strategy was more sensitive, accurate, and specific than the non-targeted approach, which was more global and systemic.

Based on our results, we hypothesize that the non-targeted metabolomics approach is advantageous for the identification of additional differentially regulated metabolites, while the targeted approach is more quantitative and can provide better selectivity.

### Systemic metabolic changes in the serum of lung cancer patients

The major metabolic alterations detected in lung cancer patients included an increase in serum ketone bodies (acetoacetate and β-hydroxybutyrate) and lactate, as well as decreased levels of glucose (β-glucose and α-glucose), lipids, unsaturated lipids, choline phospholipid metabolites (glycerophosphocholine, phosphocholine, and choline), TMAO, and betaine. Most amino acids including glutamine, glutamate, asparagine, aspartate, tyrosine histidine, cysteine, isoleucine, and leucine were elevated in the serum of lung cancer patients. In contrast, the levels of tryptophan and methionine were reduced in these patients.

As expected, a significant decrease in serum glucose and an increase lactate were observed in lung cancer patients. Cancer cells preferentially maintain a high rate of aerobic glycolysis even in the presence of adequate oxygen. This leads to increased lactate production, which is known as the “Warburg effect”. The “Warburg effect” is a common phenomenon in a variety of tumors [25]. Cancer cell growth is energy-dependent. Therefore, the most obvious explanation for increased glycolysis in cancer metabolism is a rapid requirement for ATP. This is particularly evident under hypoxic conditions [26].

Lipid dysregulation is another common metabolic alteration in cancer. Lipid metabolism contributes to the regulation of many cellular processes such as growth, proliferation, differentiation, survival, and apoptosis. Lipid metabolism can alter the composition and permeability of the cell membrane, which can promote disease development and progression (including carcinogenesis) [27, 28]. Consistent with previous studies, the levels of serum lipids including low density lipoprotein (LDL), very low density lipoprotein (VLDL), and unsaturated lipids were reduced in lung cancer patients. This may be explained by the fact that cancer cells required excess lipids for growth, proliferation, redox homeostasis, invasion, and metastasis.

Decreases in lipid levels in blood were accompanied by increases in the levels of two ketone bodies (β-hydroxybutyrate and acetoacetate) in lung cancer patients. The levels of these ketone bodies can increase when acetyl-CoA derived from lipid β-oxidation exceeds the capacity of the TCA cycle [29]. This could also explain the decreased lipid levels and increased β-hydroxybutyrate and acetoacetate levels in the serum of lung cancer patients.

Abnormal choline phospholipid metabolism has been implicated in carcinogenesis and tumor progression. Alterations in choline phospholipid metabolism have been observed in many types of cancer [30, 31]. Choline participates in methylation reactions following oxidation to betaine (trimethylglycine), which is not only essential for the methionine/homocysteine cycle, but also plays a central role in choline-mediated one-carbon metabolism (it donates a methyl group for homocysteine remethylation to generate methionine and dimethylglycine). Choline can also undergo catabolism by intestinal bacteria to produce TMA (trimethylamine), which is further converted into TMAO [32]. Tumors usually exhibit altered choline phospholipid metabolic profiles compared to normal tissue, which are characterized by abnormally high levels of choline-containing compounds. This ultimately results in lower levels in blood. Consistent with previous studies, our data demonstrated lower levels of choline-containing metabolites including choline, phosphocholine, and glycerophosphocholine in the serum of lung cancer patients compared to healthy controls. Lung cancer patients also had lower levels of betaine and TMAO compared to controls. These results suggested that aberrant choline and one-carbon metabolism may contribute to carcinogenesis.

The choline and one-carbon metabolism pathways intersect upon generation of methionine from homocysteine (Figure 4), which is then degraded to cysteine in the transsulfuration pathway. Methionine is an essential amino acid that has a critical role in cellular metabolism. It is a precursor to S-adenosylmethionine, which provides a key source of methyl groups in the cell [33, 34]. We detected lower methionine and higher cysteine levels in lung cancer patients compared to controls. These data provide evidence for aberrant one-carbon metabolism and increased homocysteine-methionine conversion in lung cancer patients.

In addition to glucose, glutamine is essential for cancer cell proliferation and survival. It is involved in anaplerosis as well as protein, lipid, and nucleotide synthesis. The requirement for glutamine depends on the energy demands of the cancer cells. The metabolism of glutamine begins with conversion of glutamine to glutamate by glutaminase or other amidases. Deamination of glutamate yields α-ketoglutarate, an intermediate in the TCA cycle. Glutamine thereby acts as an anaplerotic substrate and supports cell survival. Glutamine can be converted into aspartate, which in turn forms oxaloacetate, malate, and pyruvate through the TCA cycle [35, 36]. Aspartate is a biosynthetic precursor to asparagine, which is used for protein synthesis. We found that lung cancer patients had higher circulating concentrations of glutamine and glutamate compared to healthy controls. Additionally, the levels of the two nonessential amino acids produced from oxaloacetate (aspartate and asparagine) were elevated in lung cancer patients compared to controls. These data suggested that glutaminolysis (or glutamine catabolism) and aerobic glycolysis were increased in lung cancer patients.

Our results demonstrated that the concentrations of circulating leucine and isoleucine were higher in lung cancer patients than healthy controls. Branched chain amino acids (BCAAs) have been associated with several types of cancer. These include isoleucine, leucine, and valine, which can regulate various signaling pathways such as protein synthesis, lipid synthesis, cell growth, and autophagy [37]. BCAAs are predominantly catabolized in skeletal muscle, which has high aminotransferase activity. The catabolism of BCAAs is important for amino acid synthesis (e.g. glutamine and alanine) [38]. Thus, upregulation of leucine and isoleucine in lung cancer patients could be explained by the energetic and proliferative needs of both the host and the tumor.

We observed lower tryptophan levels in lung cancer patients compared to healthy controls. In cancer cells, high 2,3-dioxygenase activity results in increased conversion of tryptophan into kynurenine. This creates an environment that facilitates tumor growth through inhibition of T-cell proliferation and results in T-cell apoptosis and immune tolerance [39, 40]. Thus, metabolism of tryptophan via the kynurenine pathway may play a role in lung cancer etiology [41].

Lung cancer patients exhibited higher levels of tyrosine and histidine compared to healthy controls. Tyrosine is an important precursor to catecholamine neurotransmitters (dopamine, norepinephrine, and epinephrine), thyroid hormones, and melanin. It also promotes lipid metabolism. Histidine is a semi-essential amino acid and precursor of the neurotransmitter histamine. Histamine can induce cell proliferation and differentiation, and regulates both gastrointestinal function and the immune response. Our data indicate that impaired tyrosine and histidine metabolism results in an increase in the serum levels of these two amino acids in lung cancer patients.

Our study had several limitations. First, the sample size was limited. Therefore, confirmatory studies are necessary. Since patients are generally diagnosed with advanced-stage lung cancer, stage I lung cancer serum samples are difficult to collect. We collected serum samples from patients with adenosquamous carcinomas, adenocarcinomas, and small cell lung carcinomas, and aimed to identify early multilevel markers of different lung cancer subtypes. In our ongoing work, we are focusing on each specific subtype and are collecting specimens from a larger cohort of patients. The study was also limited in that the RRLC-based targeted metabolomics approach restricted the panel of candidate markers and only focused on amino acid metabolic pathways. Therefore, we are currently establishing lipid-, sugar-, and fatty acid-based metabolomics approaches in order to test and verify the feasibility of targeted metabolomics strategies. Histidine and tyrosine were identified as differentially regulated metabolites based on the ^1^H NMR-based non-targeted metabolomics approach. However, these residues were not identified using the RRLC-based targeted metabolomics approach. This is because they had VIP values < 1 in the targeted approach. An increase in both amino acids was detected in lung cancer patients using the two metabolomics approaches (Supplementary Table S1). This is the major limitation of the ^1^H NMR-based non-targeted metabolomics approach. Because of the relatively poor sensitivity, analysis of low-abundance metabolites is difficult.

## MATERIALS AND METHODS

### Chemicals and reagents

Acetonitrile and methanol (High Performance Liquid Chromatography grade) were purchased from Merck. Deuterium oxide (D_2_O, 99.9%) and L-amino acids were purchased from Sigma-Aldrich. The 2,4-Dinitrofluorobenzene (DNFB, analytical grade reagent) was purchased from Alfa Aesar. Ultrapure water was filtered using a Milli-Q water purification system.

### Clinical sample collection

The study protocol was approved by the Ethics Committee of Shijiazhuang Huaguang Traditional Chinese Medicine Tumor Hospital and was performed in accordance with the Code of Ethics of the World Medical Association (Declaration of Helsinki) for experiments involving humans (http://www.wma.net/en/30publications/10policies/b3/index.html). Written informed consent was obtained from all patients. Patients with stage I lung cancer who were treated at Shijiazhuang Huaguang Traditional Chinese Medicine Tumor Hospital and matched healthy volunteers were enrolled in the study. Cancer staging and classification were performed according to the American Joint Committee on Cancer (AJCC) and the updated Tumor Node Metastasis (TNM) cancer staging system of the International Union for Cancer Control (UICC). All subjects fasted overnight. Blood samples were collected from the antecubital vein in the morning pre-prandial. The samples were incubated at room temperature for 30 minutes to allow complete coagulation. Samples were then centrifuged at 3,000 rpm for 10 min at 4°C. The supernatants (serum) were collected and frozen at −80°C until analysis.

### Non-targeted metabolomics analysis using ^1^H NMR

#### ^1^H NMR

Serum samples from patients with stage I lung cancer and matched healthy controls were thawed and homogenized. A total of 200 μL D_2_O was added to 400 μL of serum and the mixture vortexed for 1 minute. The mixture was then centrifuged at 12,000 rpm for 5 min at 4°C. Finally, 550 μL of supernatant was transferred to 5 mm NMR tubes for ^1^H NMR analysis.

The ^1^H NMR experiments were performed using a Bruker AVANCE 600 spectrometer (Bruker BioSpin GmbH, Rheinstetten, Germany) equipped with a 5 mm TCI cryogenic probe and a 60 slot auto-sampler. One-dimensional ^1^H NMR spectra of all samples were acquired at 298 K using the Carr-Purcell-Meiboom-Gill (CPMG) spin echo pulse sequence with water pre-saturation to suppress the water signal and a fixed total spin-spin relaxation delay time (2nτ) of 210 ms to filter out signals from proteins and other macromolecules. The sample sequence was random. A total of 64 transients were collected into 32,000 data points for each sample. The metabolite assignments for the ^1^H NMR spectra were verified and confirmed by 2D ^1^H-^1^H total correlation spectroscopy (TOCSY) and correlation spectroscopy (COSY).

#### Data processing

The ^1^H NMR free induction decays (FID) were processed using the MestReNova NMR Suite software package (Ver. 6.0.2, Mestrelab Research, S.L., Spain). They were zero filled to 64 K and multiplied by a line broadening of 0.3 Hz to improve the signal-to-noise ratio before Fourier transformation. Both phase and baseline corrections were then performed manually, and the spectra were referenced to the methyl doublet signal of lactate (δ 1.33 ppm). The spectra between δ 9.0–0.0 ppm were data-reduced to 450 consecutive non-overlapped regions (bins) with an equal width of 0.02 ppm. The region between δ 5.1–4.7 ppm (containing the residual peak from the suppressed water resonance) was excluded from further analysis. The remaining bins for each spectrum were integrated and normalized according to the total area of the spectrum. The resulting normalized datasets were saved in text format and subjected to multivariate analysis.

### Targeted metabolomics analysis by RRLC

RRLC-based targeted metabolic profiling analysis of SFAAs was performed using an Agilent 1260 Series Rapid Resolution LC (Agilent technologies, Waldbronn, Germany). An Agilent Zorbax Eclipse Plus C18 (4.6 mm × 50 mm, 1.8 μm) column was used for the analysis. The analysis was performed as described previously [24]. The raw data files were processed using the *Agilent* OpenLAB Control Panel software (Version A.01.02). Prior to multivariate analysis, we measured the concentrations of SFAAs (μmol·L^−1^ serum) in patients with lung cancer and healthy controls.

### Multivariate statistical analysis

Normalized metabolomics datasets were imported into the SIMCA-P version 12.0 software package (SIMCA-P^+^ 12.0, Umetrics, Umeå, Sweden) for multivariate analysis. Principal component analysis (PCA) and orthogonal projection on latent structure discriminant analysis (OPLS-DA) were applied with mean-centering and unit-variance scaling [42]. Parameters including the cumulative values of the total Y explained variance (R^2^) and the Y predictable variation (Q^2^) were analyzed to ensure the quality of the multivariate models. Permutation tests with 100 iterations using the 7-fold cross-validation method were performed to avoid the risk of over-fitting [43].

### Selection and identification of differentially regulated metabolites

Differentially regulated metabolites that discriminated between lung cancer patients and healthy controls were obtained by variable importance in projection (VIP, a measure of their relative influence on the model) with a threshold > 1 in the OPLS-DA model. They were validated at the univariate level using student's *t*-tests (*p* < 0.05). Each metabolite was normalized and plotted in a histogram using Origin version 8.0. External cross-validation was performed to evaluate the predictive and diagnostic accuracy of the differentially regulated metabolites. Eight samples from each group were randomly selected as a test set. The remaining samples comprised the training set, which was used for validation. We used the training set to generate a prediction model that was used to predict diagnoses in the test set.

## CONCLUSIONS

Both the non-targeted and targeted metabolomics approaches could differentiate between serum samples from patients with lung cancer and healthy controls. This data indicates metabolomics analysis could be used to detect early metabolic changes associated with lung cancer progression, and could be used to detect early-stage lung cancer. Many metabolites were significantly altered in lung cancer patients compared to healthy controls. These included glycolysis metabolites, lipid metabolites, phospholipid intermediates, ketone bodies, and amino acids. Collectively, our data demonstrate the feasibility of our approach. However, additional studies involving larger patient cohorts are required to validate the clinical significance of the non-targeted and targeted metabolomics approaches for cancer detection.

## SUPPLEMENTARY MATERIAL AND TABLE


