# Supplementary material for: Non-targeted and targeted metabolomics approaches to diagnosing lung cancer and predicting patient prognosis
Source: Oncotarget. 2016 Aug 23;7(39):63437–48. doi: 10.18632/oncotarget.11521 (PMC5325375; doi:10.18632/oncotarget.11521)
Supplement: Supplementary file 1 [file oncotarget-07-63437-s001.pdf]

Non-targeted and targeted metabolomics approaches to diagnosing lung cancer and predicting patient prognosis

Supplementary Materials

Supplementary Table S1: Concentrations of 23 serum free amino acids in lung cancer patients and healthy controls[a]

Amino acids	Concentrations (μmol·L^{-1}, n = 25)	
	Healthy control	Patients with lung cancer
Aspartic acid (Asp)	14.88 ± 3.88	28.17 ± 8.55
Glutamic acid (Glu)	81.28 ± 29.77	145.81 ± 59.05
Hydroxyproline (Hyp)	11.11 ± 4.78	19.10 ± 11.38
Asparagine (Asn)	8.28 ± 4.08	29.79 ± 7.53
Glutamine (Gln)	93.39 ± 20.95	126.62 ± 17.81
Serine (Ser)	234.23 ± 45.70	202.94 ± 40.52
Glycine (Gly)	258.29 ± 44.50	241.24 ± 34.76
Arginine (Arg)	9.75 ± 1.77	11.08 ± 3.32
Threonine (Thr)	67.91 ± 9.35	58.22 ± 18.25
Histidine (His)	51.80 ± 8.21	68.11 ± 19.34
Taurine (Tau)	76.23 ± 17.97	79.55 ± 14.62
Alanine (Ala)	374.07 ± 65.98	372.26 ± 90.41
Proline (Pro)	154.90 ± 47.58	163.16 ± 32.46
Valine (Val)	244.80 ± 48.52	208.41 ± 32.35
Cysteine (Cys)	13.48 ± 5.93	25.46 ± 7.48
Methionine (Met)	14.62 ± 3.94	7.74 ± 0.66
Ornithine (Orn)	88.20 ± 14.19	74.63 ± 14.73
Isoleucine (Ile)	102.27 ± 21.53	157.18 ± 19.38
Tryptophan (Trp)	41.37 ± 13.07	19.38 ± 3.47
Leucine (Leu)	59.07 ± 7.42	117.35 ± 18.48
Phenylalanine (Phe)	43.95 ± 6.74	60.21 ± 24.40
Lysine (Lys)	69.72 ± 11.16	93.58 ± 25.72
Tyrosine (Tyr)	56.69 ± 9.09	77.22 ± 28.55

[a]The concentrations are presented as mean ± standard deviation.
